# Barriers and facilitators to using aspirin for preventive therapy: a qualitative study exploring the views and experiences of people with Lynch syndrome and healthcare providers

**DOI:** 10.1186/s13053-022-00235-z

**Published:** 2022-08-23

**Authors:** Kelly E. Lloyd, Robbie Foy, Louise H. Hall, Lucy Ziegler, Sophie M. C. Green, Zainab F. Haider, David G. Taylor, Mairead MacKenzie, Samuel G. Smith

**Affiliations:** 1 Leeds Institute of Health Sciences, University of Leeds, Clarendon Way, Leeds, LS2 9NL UK; 2 School of Pharmacy, University College London, London, UK; 3 Independent Cancer Patients’ Voice, London, UK

**Keywords:** Preventive therapy, Chemoprevention, Decision-making, Lynch syndrome, Aspirin, NSAID

## Abstract

**Background:**

The National Institute for Health and Care Excellence (NG151) recommends considering daily aspirin for people with Lynch syndrome to reduce colorectal cancer risk. However, deciding whether to initiate aspirin could be a complex decision for patients and their healthcare providers, as both the potential benefits and harms need to be considered.

**Methods:**

We conducted semi-structured interviews to explore the barriers and facilitators to using aspirin for preventive therapy. We recruited 15 people with Lynch syndrome, and 23 healthcare providers across multiple professions in primary, and specialist care (e.g. clinical genetics) in the United Kingdom. Interview schedules were informed by the Theoretical Domains Framework.

**Results:**

There were three themes: 1) Considering potential harms and benefits; 2) Healthcare pathway; 3) Patients’ level of interest in aspirin. All healthcare providers, across primary and specialist care, viewed general practitioners (GPs) as being responsible for prescribing and overseeing the use of aspirin. However, GPs were unfamiliar with aspirin for preventive therapy, and concerned about prescribing at higher doses (300-600 mg). To support decision-making, GPs wanted clarification from specialist clinicians on the evidence and dose to prescribe. Not all participants with Lynch syndrome received information on aspirin from their healthcare provider, and several were unsure who to discuss aspirin with. GPs were more inclined to prescribe aspirin for patients with expressed preferences for the medication, however several patients were uncertain and wanted further guidance.

**Conclusions:**

Coordinated and multilevel strategies are needed, addressing the needs of both GPs and people with Lynch syndrome, to ensure consistent implementation of national guidance on aspirin for preventive therapy.

**Supplementary Information:**

The online version contains supplementary material available at 10.1186/s13053-022-00235-z.

## Introduction

Lynch syndrome (LS) is an inherited disorder caused by faulty mismatch repair genes (MLH1, MSH2, MSH6, PMS2) [1]. People with LS have an increased risk of developing a spectrum of cancers, including colorectal cancer [2, 3], with studies estimating a 10–46% lifetime risk of colorectal cancer, depending on gender and the mismatch repair gene affected [2, 4]. Aspirin has been investigated as a potential preventive therapy agent for colorectal cancer. The CAPP2 trial found participants with LS randomised to receive aspirin at 600 mg daily (vs. placebo) for at least 2 years had a reduced risk of developing colorectal cancer at 10 year follow-up (hazard ratio of 0.65 in intention-to-treat analysis) [5]. A dose non-inferiority trial (CaPP3) is currently underway to compare the effectiveness of aspirin at different doses (100 mg, 300 mg, or 600 mg) for colorectal cancer prevention. At present, the evidence for a preventive effect of aspirin on non-colorectal LS cancers is weak [5]. In the United Kingdom (UK), the National Institute for Health and Care Excellence (NICE) updated their colorectal cancer guideline (NG151) in 2020 with a recommendation to consider daily aspirin to reduce the risk of colorectal cancer in people with LS [6]. The guidance does not stipulate a dose, but 150-300 mg is commonly used in practice [6].

Deciding whether to initiate preventive therapy can be a complex choice for patients. In the area of breast cancer prevention, women at higher risk of breast cancer express reluctance to initiate preventive therapy using tamoxifen due to concerns regarding side-effects [7–10], and perceived lack of control over their cancer risk [8]. The facilitators and barriers patients experience when considering the use of aspirin for preventive therapy have been less explored [11]. People with LS need to consider both the risks and benefits of aspirin for cancer prevention. While there are demonstrable benefits, even low doses of aspirin can increase the risk of gastrointestinal ulceration and bleeding [12], with these risks increasing substantially after the age of 70 [13]. At present, the NICE guidance NG151 does not specify an age limit for the long term use of aspirin among people with LS. However, it does stipulate that aspirin may not be suitable for particular cases, such as people with a history of peptic ulcers [6].

It is also important to consider the perspectives of healthcare providers when implementing clinical guidance. At present, the NICE guidance does not specify a recommended healthcare prescriber [6]. Previously, the introduction of cancer preventive therapy within specialist care has led to uncertainties with regard to prescribing responsibilities [14]. An Australian interview study explored healthcare providers’ (e.g. specialists, pharmacists, general practitioners (GPs)) views on the use of aspirin for colorectal cancer prevention in the general public [15]. Healthcare providers described multiple barriers to recommending aspirin, including confusion over which dose to prescribe and concerns about side-effects, especially in older populations [15]. In addition, GPs were viewed as the most important healthcare provider for implementing the Australian guidance recommending aspirin. Qualitative research exploring the views of healthcare providers on the use of aspirin for cancer prevention in a LS population has not yet been undertaken [11]. However, a cross-sectional survey of UK GPs observed respondents were more willing to prescribe aspirin to a person with LS if they had greater awareness of its cancer preventive effects [16]. Furthermore, the dose of aspirin influenced willingness to prescribe, with only 62% of GPs willing to prescribe daily aspirin at 600 mg compared with 91% at 100 mg [16].

Here, we conducted qualitative interviews to explore the perceived or experienced barriers and facilitators to using aspirin for preventive therapy among people with LS. We also explored the perceived or experienced barriers and facilitators to prescribing or recommending aspirin among healthcare providers involved in the LS healthcare pathway, including perspectives on the NICE guidance (NG151).

## Method

### Design

We conducted semi-structured one-to-one interviews with participants. The study was pre-registered (https://osf.io/3efg7).

### Participants and recruitment

We recruited both people with LS and healthcare providers, with recruitment organised by one author (KEL) and supported by co-authors. Across both participant groups, people based in the UK and over the age of 18 were recruited. We advertised the study through the charity Lynch Syndrome UK (LSUK), aiming to recruit both people with LS who use and do not use aspirin for prevention. People who had not been diagnosed with LS were excluded. To recruit healthcare providers, we used snowball sampling, and advertised the study through social media (e.g. Twitter) and relevant professional organisations. We recruited healthcare providers involved in the LS healthcare pathway, including GPs, community pharmacists, genetic counsellors, nurse practitioners, and specialist clinicians. Specialist clinicians included those in the roles of clinical geneticist, consultant in cancer genetics, gastroenterologist, and gynaecologist. Healthcare providers were excluded if their clinical roles did not appear to include potential discussions with people with LS about aspirin. All participants received a £25 Amazon voucher.

One author (KEL) recruited participants in both groups until data saturation had been reached, and followed an established method to assess this [17]. Initially a minimum sample size of 10 was sought in each participant group before looking for evidence of data saturation [17]. After 10 interviews had been conducted, data saturation was assessed. Data saturation was judged to have been achieved for each group once three further consecutive interviews had been conducted which yielded no new themes. For example, recruitment would cease after interview 13, if interviews with participants 11, 12, and 13 resulted in no new themes.

### Interview schedule

At the beginning of all interviews, the NICE guidance (NG151) recommending aspirin for colorectal cancer to people with LS was described. We presented participants with basic information on aspirin for two main reasons. Firstly, to create a more realistic clinical scenario where participants would consider using or recommending aspirin in relation to existing information, such as official guidance, dose and duration. Secondly, we did not want the interviews to be perceived by participants as a test of their prior knowledge on the use of aspirin for preventive therapy.

A semi-structured interview approach was employed, with improvised follow up questions guided by participants’ responses, and with flexibility to the order of the questions asked. The interview schedule covered the 14 domains in the Theoretical Domains Framework (TDF; version 2) (Supplementary Materials) [18]. The TDF is a theoretical framework derived from multiple behaviour change theories. The framework identifies several factors (i.e. domains) that could influence behaviour when implementing new clinical practices, such as a person’s knowledge, skills, beliefs, environment and the resources available to them [18]. This framework was chosen as it has previously been used to explore influences on healthcare provider and patient behaviour when implementing evidence-based recommendations [18–20]. The draft interview schedule was reviewed by a patient representative to assess for comprehension before finalising (MM).

In the healthcare provider interviews, we also presented participants with clinical vignettes, with the aim to explore the potential barriers to recommending aspirin among healthcare providers who may not have experience in this area. We developed realistic scenarios where the participant may encounter a patient with LS enquiring the use of aspirin for colorectal cancer prevention. The scenarios were reviewed by a primary care clinician before finalising (RF). We explored healthcare providers’ initial thoughts and likely responses to these scenarios.

### Data collection and analysis

One author (KEL) conducted all interviews, over video or telephone, from November 2020 to November 2021. KEL is a behavioural scientist with academic training in qualitative research methods. She had not previously undertaken qualitative interviews, but was supported by a team of experienced investigators who met with her regularly throughout the recruitment period. Interviews were audio-recorded, transcribed verbatim, and anonymised. All participants were given pseudonymised initials.

Our two-stage analysis involved coding the transcripts inductively using reflexive thematic analysis [21, 22], and mapping the extracted themes onto the TDF [18]. We mapped our themes onto the TDF as this framework can aid in specifying the beliefs and attitudes that are amenable to change [18]. In turn, this can inform strategies to implement aspirin for preventive therapy into clinical practice. In addition, we employed the TDF flexibly alongside an inductive analysis approach, which can help to identify themes and factors unrelated to the TDF [23]. One author (KEL) coded all transcripts, while three additional authors (SGS, SMCG, ZFH) double-coded a proportion of transcripts. All four authors discussed the findings and reached consensus on the final themes. One author (KEL) mapped the themes onto the TDF, which was reviewed by all authors. Transcripts were managed in NVivo (version 12) and Microsoft Word.

## Results

We interviewed 15 people with LS (Table 1), and 23 healthcare providers across multiple disciplines (Table 2). Interview duration ranged from 22 to 60 min. Findings were organised into three overarching themes, which were mapped onto the TDF (Table 3).

**Table 1:** Description of the people with LS interviewed (*n* = 15)

	***n***
**Age**	
18–30	0
31–40	1
41–50	5
51–60	5
61–70	4
**Gender**	
Male	2
Female	13
**Ethnicity**	
White British	13
White British and Irish	1
White European	1
**Country in UK**	
England	12
Scotland	2
* Missing data*	1
**Year of Lynch syndrome diagnosis**	
1990–2000	2
2001–2011	0
2012–2021	13
**Previously diagnosed with cancer**	
Yes	9
No	6
**Using aspirin for preventive therapy**	
Yes	9
No	6

**Table 2:** Description of the healthcare providers interviewed (*n* = 23)

	***n***
**Age**	
18–30	6
31–40	7
41–50	4
51–60	3
61–70	2
* Missing data*	1
**Gender**	
Male	7
Female	16
**Ethnicity**	
White British	16
White European	2
British Asian/ Asian	3
Black Caribbean	1
* Missing data*	1
**Country in UK**	
England	20
Wales	3
**Profession**	
General practitioner	9
Community pharmacist	4
*Specialists*	
Genetic counsellor / nurse practitioner	5
Specialist clinicians	5
**Number of years in profession**	
0–10	14
11–20	3
21–30	4
31–40	2
**Previously encountered patient with LS**	
Yes	13
No	10
**If yes, approximately often do you encounter a patient with LS**	
Daily	3
Weekly	3
Monthly	3
Once or twice a year	4

**Table 3:** The themes, and corresponding facilitators, barriers, and domains within the Theoretical Domains Framework (TDF; version 2)

Themes	Potential facilitators to the use of aspirin for preventive therapy	Potential barriers to the use of aspirin for preventive therapy	Main TDF domain(s)
Considering potential harms and benefits	Confidence in the evidence supporting aspirin for colorectal cancer prevention National guidance (i.e. NICE) recommending aspirin for preventive therapy Low concerns about using aspirin as it is a pharmacy drug	Concerns about using daily aspirin at higher doses (300-600 mg) Lack of strong evidence to support an appropriate dose of aspirin which balances the benefits and harms	Beliefs about consequences
Healthcare pathway	Agreement among GPs and specialists on the appropriate healthcare pathway for patients to acquire a prescription for aspirin	Most GPs are unfamiliar with evidence supporting the use of aspirin for colorectal cancer prevention Lack of clarity on the appropriate treatment pathway for aspirin among people with LS Specialist clinicians in genetics may be an underutilised resource among GPs Some people with LS may be reluctant to approach their GP to discuss aspirin	Social/professional role and identity Environmental context and resources Knowledge
Patients’ level of interest in aspirin	Patients having a high level of knowledge on the risks and benefits of aspirin Patients’ expressed preference to use aspirin	Patients who are uncertain whether to use aspirin and require further support	Knowledge Environmental context and resources

*Note.* Table adapted from Burgess et al. [24]

### Considering potential harms and benefits

#### Consideration of benefits

Participants considered the benefits of aspirin and the evidence supporting this recommendation in their decision-making. Participants with LS typically had high confidence in the evidence supporting the use of aspirin for preventive therapy. Among healthcare providers, confidence in the evidence varied. Specialists were positive about using aspirin, while GPs and community pharmacists tended to be more sceptical.

“I think it’s amazing that there is a drug that is so cheap and with lots of safety data and been used for 100 years that has a demonstrable and significant effect on cancer prevalence in Lynch syndrome.” (S.D., specialist clinician, 0-10 years’ experience)

“So the answer is, at the moment, for me the jury’s still out and it sounds like it is from the latest studies as well.” (H.H., GP, 31-40 years’ experience)

Although GPs were unaware prior to the interview of the NICE guideline NG151, this organisational body was considered to be a trustworthy source. Learning of the NICE recommendation appeared to increase several GPs’ confidence in the effectiveness of aspirin for preventive therapy and their comfort prescribing aspirin for this purpose.

“You know, and I’m kind of thinking if someone came in, I’d kind of think god, if it’s in NICE guidelines I’d kind of very much believe it.” (T.Y., GP, 0-10 years’ experience)

#### Consideration of harms

Several participants with LS and healthcare providers discussed aspirin’s adverse effects as an important barrier to using or recommending aspirin. In considering dose, participants with LS were more worried about using higher doses of aspirin, such as 300 mg or 600 mg, because of potential harms.

“There’s no way I would take that 300, oh my god, no, if I was having as many problems with 150.” (L.O., participant with LS)

Among healthcare providers, GPs and community pharmacists in particular expressed concerns about patients using aspirin at higher doses, due to the increased risk of side-effects such as gastrointestinal bleeding.

“I think I’d be less hesitant if it was a lower dose medication such as 75 or 150mg, I’m clinically comfortable with. You know, 600mg doses is not something I’m used to prescribing, […] So I’d be worried about their bleeding risk, especially if they were elderly and frail.” (F.F., GP, 0-10 years’ experience)

“You know, my thoughts would be that 600mg would be quite a significant risk to patients at risk of GI issues.” (B.K., community pharmacist, 0-10 years’ experience)

Across both groups, not all participants considered the risks of aspirin to be a prominent factor in their decision-making, partly because aspirin is a well-known medication that can be purchased from pharmacies without a prescription.

"People sort of take aspirin a bit like, you know, paracetamol. So, so many millions of people have taken it that it seems that the side-effects that you might possibly get would be minimal." (Z.B., participant with LS)

“I think anything that a patient can happily buy over-the-counter, whatever reason, sits a little bit happier with GPs.” (F.P., GP, 0-10 years’ experience)

Most GPs discussed prescribing proton pump inhibitors (PPI) alongside aspirin for patients at higher risk of gastrointestinal ulcers and bleeding [25], which in turn lowered their concerns regarding the harms.

“I think we rarely actually see GI bleeds and things, I think we've got better at prescribing […] like Omeprazole [a PPI] you know something that’s going to reduce acid and things alongside.” (T.Y., GP, 0-10 years’ experience)

#### Considering the harms vs. benefits

Most participants with LS felt that the potential benefits of aspirin for colorectal cancer prevention outweighed their concerns about aspirin’s side-effects. This was generally supported by healthcare providers.

“My father’s had two cases of bowel cancer, and the second one it nearly killed him, I don’t want that, I don’t want bowel cancer. So yeah, for me I’ll take [aspirin] to reduce that.” (A.D., participant with LS)

“Obviously there are potential side-effects and risks but if those can be ruled out, the benefits of taking it are huge, particularly for the kind of sub-group of patients that we deal with.” (M.C., genetic counsellor/nurse practitioner, 11-20 years’ experience)

However, some patients explained how they made difficult trade-offs when deciding to take aspirin.

“I’m not particularly happy about taking aspirin […] it could trash your stomach, it could trash other parts of your body. But if it reduces your risk of cancer you feel there’s a gun to your head in a sense.” (L.O., participant with LS)

The lack of strong evidence to support an appropriate dose of aspirin which balances the benefits and harms, and the absence of a recommended dose by NICE for this reason [6], was a concern among several healthcare providers and participants with LS. Among participants who did not use aspirin, some felt that at present the risks outweighed the benefits for them.

“I find those discussions about dosing quite tricky […] we have a rough guidance of the dosing but we don’t really know exactly what that’s going to do and whether we need to change that in the future once the CaPP3 dose comes out.” (A.P., specialist clinician, 0-10 years’ experience)

“The benefits have had to outweigh the risks, but at the moment not until somebody tells me exactly how much I should be taking, I’m not going to start on [aspirin].” (R.R., participant with LS)

### Healthcare pathway

#### Perceptions of the ideal healthcare pathway

Healthcare providers across professional groups viewed specialists as patients’ main source of information regarding aspirin for preventive therapy; they were perceived as having the requisite expertise in this topic area. Healthcare providers agreed GPs were responsible for prescribing aspirin, as they will have access to patients’ medical histories to check for potential contraindications.

“I think [aspirin] would probably be kind of started in conjunction with specialist advice but then we would carry on prescribing it long-term.” (K.M., GP, 0-10 years’ experience)

“I’m primarily focusing on information giving in that appointment [with the patient] and it’s important if you are going to start a new medication that you do that in conjunction with your GP.” (A.P., specialist clinician, 0-10 years’ experience)

However, GPs were mostly unfamiliar with the evidence for using aspirin for colorectal cancer prevention, and required further support from specialist clinicians before prescribing. GPs wanted clarity on the appropriate dose to prescribe, the supporting evidence, the referring clinician’s opinion on this evidence, and a clear recommendation to prescribe.

“Well I would want to know what the recommended dose and timescale was and it would also be helpful to know a bit more about how much it reduces the risk and about what the risk reduction actually is.” (Z.E., GP, 11-20 years’ experience)

“So as long as it said please prescribe, if it said please consider prescribing then again it’s a more complicated scenario, […] It depends on what the wording is from the geneticist.” (G.H., GP, 21-30 years’ experience)

Specialists, across areas such as genetics and gastroenterology, agreed their role included supporting GPs who were considering prescribing aspirin for a patient with LS.

“I see lots of patients with Lynch, so I feel it’s a decision in the sense that we’re better placed to make and I feel it’s only fair that I could give the GP as much guidance as I can in that.” (O.I., specialist clinician, 0-10 years’ experience)

#### Healthcare pathway in practice

In reality, pathways to treatment were inconsistent. Despite specialists accepting their role as information providers, not all participants with LS were told about aspirin in a healthcare setting. Some participants first learnt about aspirin through other sources, such as the charity LSUK.

“Yeah, I have actually [been told about aspirin], not through the hospital that I’m under, or like my GPs or anything, mainly […] from joining the [LSUK] side.” (B.H., participant with LS)

Although clinical geneticists viewed their role as providing information on aspirin, not all GPs made use of this source. Instead, several GPs were more likely to approach the patient’s colorectal cancer team for discussions.

“Well you’ve even also got local sources, so we get access to individual colorectal teams, for instance. […] We might occasionally use genetics but I haven’t used a geneticist for yonks really, so I couldn’t say hand on heart that I would use them straightaway.” (H.H., GP, 31-40 years’ experience).

Several participants with LS found the pathway unclear. They were unsure which type of healthcare provider they should approach to discuss aspirin further with, and where they should acquire the medication from.

“I mean, my first instinct would just be go to the pharmacy and buy it but I don’t know what the dose is that you get there […] so I guess I’d try just to buy it first but if it wasn’t the right dose I guess I’d go to maybe the GP and get it prescribed.” (Z.B., participant with LS)

Not all participants with LS were aware of the option for aspirin on prescription, and instead purchased aspirin from the pharmacy. In contrast, community pharmacists felt it was not their role to sell higher doses of aspirin (> 75 mg) for preventive therapy to patients without a prescription. The lack of licence for this indication was a particular issue for this group.

“I couldn’t imagine it getting to the point where we’d be […] selling aspirin over the counter for that indication, […] it would be off-label use.” (B.K., community pharmacist, 0-10 years’ experience)

Equally, several participants with LS were recommended by a specialist clinician to approach their GP for aspirin on prescription and had obtained the medication through this route.

“My GP actually prescribed the aspirin and they never sort of questioned it, […] they just said, ‘oh well if it’s been recommended by the geneticist, fine we’ll do it’.” (T.R., participant with LS)

However, not all participants were comfortable approaching their GP to discuss aspirin, due to previous negative experiences with GPs who were unfamiliar with LS.

“I find that the GPs aren’t very clued up about Lynch syndrome. […] So no, I don’t find going to the GPs very useful, unfortunately.” (Z.B., participant with LS)

### Patients’ level of interest in aspirin

There was a strong interest in using aspirin among participants with LS currently using the medication. These participants typically considered aspirin a high priority, and were motivated to research the use of aspirin for preventive therapy and the recommended dose.

“So I gathered all the information, read all the information, had a look around, went onto the [LSUK] site […] so I did a lot of research into it, and basically sort of discovered really that I should be on about 300mgs aspirin a day.” (A.D., participant with LS)

Using aspirin for preventive therapy appeared to be a lower priority among participants who did not use the medication, especially when compared with other life and family priorities. Furthermore, other preventive options for LS seemed to be considered higher priorities, or more effective options, such as surgery and surveillance.

“I wasn’t actually given any other [information from GP surgery] than ‘oh well there’s not a lot of research that shows it’s kind of very beneficial’ […] I mean, I could’ve like researched and everything in the meantime but as I say, life gets in the way.” (K.J., participant with LS)

“Well I suppose I’m not so worried about my bowel cancer coming back because I would just have the lot removed, […] and I think it would be picked up before it could do me any damage.” (H.A., participant with LS)

A patient’s strong interest in aspirin was an important factor for GPs. Several GPs described feeling more inclined to prescribe aspirin, especially higher doses, for patients who were already keen to use the medication and appeared knowledgeable on the subject.

“If the patient really wanted to start it, they’ve done the research, they understand the risks and benefits then yeah, I probably would feel comfortable [prescribing aspirin].” (M.V., GP, 0-10 years’ experience)

The tendency to be more willing to prescribe aspirin for patients who have already decided to use the medication may be problematic, as several participants with LS were uncertain and wanted further guidance. In particular, some participants wanted a clear recommendation to use aspirin from their healthcare provider, based on such factors as their medical history.

“I want somebody to tell me you know, yes this would be ideal for you, or to say no, because you’ve got this, […] rather than it just be my decision." (R.R., participant with LS)

The relationship between patients’ prior preferences for aspirin and acquiring a prescription is further illustrated by two individuals. Participant A.D., who wanted to use aspirin at 300 mg, described how they encountered recurrent barriers before they acquired a prescription at this dose.

“ [GP] rang up and said, “Yes, you can have it on prescription,” and went and had a look at it, and basically it was for 75mgs, and so I went back to see him and I said, “This really isn’t, you know, enough,” […] And then finally after probably a good couple of months going backwards and forwards he agreed that I could take 300mgs of aspirin a day.” (A.D., participant with LS)

Participant K.J., who was more uncertain, encountered resistance from their GP surgery and subsequently did not initiate aspirin.

“When I then contacted my GP surgery to get a prescription for that I was kind of put off getting it, probably thinking about it now due to their lack of knowledge.” (K.J., participant with LS)

## Discussion

In this interview study, both people with LS and their GPs were found to have a range of unmet informational needs around the use of aspirin for preventive therapy, which are inconsistently supported by current treatment pathways. GPs were seen by all healthcare providers, across primary and specialist care, as the main prescribers of aspirin. However, GPs were unfamiliar with the use of aspirin for colorectal cancer prevention, and wanted clarification from specialists on the evidence and dose to prescribe. Furthermore, there were varying levels of support for people with LS considering aspirin. For example, not all participants with LS received information on aspirin from their healthcare provider, and several were unsure who to discuss aspirin with.

Our findings are consistent with previous healthcare provider research conducted in Australia [15] and the UK [16], which identified several barriers to prescribing aspirin among GPs. These barriers included low awareness of the national guidance recommending aspirin for cancer prevention, and concerns regarding the side-effects of aspirin at higher doses. In our study, we compared and contrasted perspectives of both patients and healthcare providers to develop a more complete understanding of areas for improvement than if we had focused on one group. For example, our study adds further to the literature by demonstrating that patients with LS also have concerns about using aspirin at higher doses, which may subsequently affect whether they initiate preventive therapy.

Our results indicate that a shared decision-making approach could be valuable for patients who are uncertain on whether to initiate aspirin and want further guidance from their GP, with the aim for both parties to reach consensus and agreement on the decision [26]. Where clinical evidence is uncertain, recommended approaches to promoting shared decision-making include tailoring information to the needs of the patient, and utilising decision support technology (e.g. decision aids) [27]. However, any such shared decision-making approaches need to be adaptable to the realities of clinical practice. For example, a UK study found that primary care consultations covered an average of 2.5 problems in just under 12 min [28]. Furthermore, our findings highlight that GPs alone may not have the knowledge and resources to fully support a patient considering aspirin for preventive therapy.

Our work suggests that initiatives to support shared decision-making are unlikely to bring about significant change in isolation. We found the TDF useful in understanding further how the identified barriers and facilitators to the use of aspirin for preventive therapy could inform future implementation strategies [18]. The four main TDF domains we identified were: the ‘Social/professional role and identity’ of the healthcare providers; ‘Environmental context and resources’; ‘Beliefs about consequences’ of using aspirin; and existing ‘Knowledge’ regarding the use of aspirin for preventive therapy.

The integration of aspirin for cancer prevention into clinical practice is likely to depend on clearly defined and consistently applied healthcare professional roles. Our findings suggest that specialists, such as clinical geneticists, are the main providers of information on aspirin, whilst GPs are the main prescribers. However, poorly defined care pathways may result in environmental context barriers, such as some patients with LS being unaware of the option to use aspirin for preventive therapy. There is a need for a coherent strategy, developed in collaboration with specialist and primary care, to ensure consistent and equitable support for people with LS and their GPs. Such a strategy should recognise that successful change will depend upon coordinated efforts across different levels of healthcare systems [29], with national guidance underpinned by clear local healthcare pathways and defined roles. In addition, such pathways should specify who is responsible for the assessment, counselling, and treatment of people with LS, as well as specifying how aspirin is prescribed and recorded in patient records.

Both patients and healthcare professionals may benefit from support for relatively complex decisions. Existing resources, such as the NICE patient decision aid for people with LS considering aspirin [30], should be consistently utilised in the healthcare pathway. Decision aids can improve patient knowledge of treatment options and reduce feelings of uncertainty around decisions [31]. In addition, future research could develop and evaluate interventions to support GPs when advising patients with LS on aspirin for colorectal cancer prevention. These interventions could target the beliefs and attitudes among GPs that we have identified are amenable to change.

Our study had several limitations. We recruited most healthcare providers through snowball sampling and Twitter, which could have resulted in an unrepresentative sample of participants who are particularly research active. In addition, we recruited all participants with LS through the charity LSUK. As several participants with LS first became aware of aspirin through LSUK, there may be different levels of awareness and interest in aspirin among a wider population of people with LS. Our sample of participants with LS mostly consisted of white women. In order to address potential health inequalities, further research should aim to understand the barriers and facilitators to using aspirin across all socio-demographic groups. Across all interviews we provided participants with information on aspirin from NICE guidance NG151. However, there is potential that some of the barriers and facilitators explored by participants may have been different without this prior information. Furthermore, in some cases participants may have been more inclined to respond positively regarding the use of aspirin for colorectal cancer due to the presence of the interviewer.

## Conclusions

GPs and patients with LS have multiple unmet informational needs in decisions concerning aspirin use, which are inconsistently supported by current care pathways. The implementation of national guidance therefore needs to be underpinned by clearly defined local roles and accessible information to support shared decision-making. Future research could include the development and evaluation of interventions to support GPs advising people with LS on aspirin for cancer prevention.

## Supplementary Information

**Additional file 1. **Interview schedules for Lynch syndrome and healthcare provider interviews.

## Data Availability

Public access to the interview schedules, and restricted access to a subset of anonymised interview transcripts is available from University of Leeds Research Data Repository: https://doi.org/10.5518/1097. Anonymised transcripts have only been uploaded to the repository for participants who consented to this procedure and for transcripts that could be reasonably anonymised.

## Abbreviations

GP: General practitioner
LS: Lynch syndrome
UK: United Kingdom
NICE: National Institute for Health and Care Excellence
LSUK: Lynch Syndrome UK
TDF: Theoretical Domains Framework
PPI: Proton pump inhibitor
