# Supplementary material for: Barriers and facilitators to using aspirin for preventive therapy: a qualitative study exploring the views and experiences of people with Lynch syndrome and healthcare providers
Source: Hered Cancer Clin Pract. 2022 Aug 23;20:30. doi: 10.1186/s13053-022-00235-z (PMC9396868; doi:10.1186/s13053-022-00235-z)
Supplement: Supplementary file 1 — Additional file 1. Interview schedules for Lynch syndrome and healthcare provider interviews. [file 13053_2022_235_MOESM1_ESM.docx]

# Interview schedule for Lynch syndrome interviews

Thank you for agreeing to take part. Just before we start the interview, I’d like to quickly go over some key points about your rights as a participant in this study. It is completely fine if at any point you would like to stop the interview, or if you do not want to answer a question, please just let me know. You can also withdraw from the study at any point and you do not have to provide a reason for why. Just to remind you the interview will be recorded, but anything you say to me today will be kept confidential, and if you are quoted in any of our reports your name will not be used and instead you will be given a pseudonym, which is a fake name. I will also anonymise anything identifiable, like places, you mention as well. After the interview, if you have any further questions about anything we discussed today, please feel free to contact me. I can also provide a debrief sheet at the end with website links with further information on this topic in case you are interested in reading more. Are you happy to continue?

## Lynch syndrome qualitative interviews

As you read on the information sheet, the study is aiming to understand what people with Lynch syndrome think about using aspirin for cancer prevention, including the reasons why people may or may not be willing to use it. First, before we start the interview questions, I would just like to briefly read a section of the guidelines for bowel cancer which have been adopted by NHS England. The guidance says:

“Consider daily aspirin, to be taken for more than 2 years, to prevent colorectal cancer in people with Lynch syndrome.”

Another section of the guideline states some of the side-effects of taking aspirin daily:

“Long-term use of aspirin may slightly increase the risk of bleeding.”

I will now move onto the interview questions, which will explore your thoughts on using aspirin regularly for cancer prevention. Just a reminder before we start, no prior knowledge on the use of aspirin for cancer prevention is needed or expected of you, we are just interested in hearing your initial thoughts.

| **Domains** | **Interview questions** |
| --- | --- |
| Knowledge  Enviromental context and resources  Intentions  Social influences | Had you heard of the use of aspirin for cancer prevention before this interview?  If yes, where and how did you hear about this?  If no, how would you find such information? Prompts: from what sources? Internet? A healthcare professional?  Who would you like to tell you about this information? Prompt: Any particular healthcare professionals, e.g. your GP?  *[If they are aware of aspirin for cancer prevention, then ask:]*  Do you already take aspirin regularly for cancer prevention?  *[If no]* Is there any particular reason why you don’t take it?  *[If yes]* What factors influenced your decision to use aspirin?  Have you ever been encouraged or discouraged by someone to take aspirin regularly for cancer prevention? Prompt: Why was this? |
| Optimism  Beliefs about consequences  Emotions  Enviromental context and resources | In your opinion, do you think using aspirin would be a good way to reduce your risk of developing bowel cancer in the future? Prompt: Why do you think this?  Do you have any concerns about taking aspirin regularly? Prompts: How do you feel about the side-effects?  What kind of information do you think you would need to help you make a decision on whether to take aspirin regularly for cancer prevention?  Where would you go to get this information? Prompts: What would your first step be to obtain this information? Internet? Healthcare professionals? |
| Beliefs about consequences | I mentioned before one of potential side-effects of regular aspirin use can be internal bleeding. There are several risk factors that make a person more likely to experience internal bleeding from regular aspirin use. These include:   - Active or previous peptic ulceration - Bleeding disorders - Over the age of 65 - Uncontrolled hypertension - Previous history of stroke - Abnormal liver or renal function - Experience indigestion on aspirin   If you had any of these risk factors, your doctor may offer you an additional medication to reduce your risk of internal bleeding.  What are your thoughts on taking regular aspirin alongside another medication to reduce your risk of bowel cancer? |
| Skills  Beliefs about capabilities  Emotion  Reinforcement  Enviromental context and resources | If you were interested in taking aspirin for cancer prevention, do you know how you would obtain a prescription for daily aspirin? Please describe how you would go about this. Prompts: What would your first step be?  How comfortable would you feel about going through this route to get a prescription for aspirin?  *[If they mention they already have prescription/ tried to get prescription for aspirin]*  Have you previously encountered any problems trying to get a prescription for daily aspirin? If yes, please describe these problems.  Is there anything about this experience that makes you more or less likely to take aspirin regularly in the future? |
| Goals  Memory, attention and decision processes | How much of a priority is taking aspirin for cancer prevention to you? Prompts: how high or low a priority is it  Are there any other higher priorities? Prompt: Prevention priorities? What are they? |

##

## Brief quantitative questions

Thank you so much for your answers so far. Just before we end the interview, there are several brief demographic questions that I would just like to go through with you. If you do not want to answer one these questions, please let me know and we can skip it.

1. What is your age?
2. Please describe your gender? Male; Female; Non-binary; Another identity; Prefer not to say.
3. How would you describe your ethnicity? Examples include White British, Indian, Mixed – White and Black Caribbean
4. What country in the UK do you live?
5. Year of Lynch syndrome diagnosis?
6. Have you previously been diagnosed with cancer?

## Debrief

Thank you for all your help with answering my questions. We really appreciate your time and hope that it will be useful in the future when we are trying to support people when making a decision about whether to use aspirin for cancer prevention. Before we end, do you have any questions you would like to ask me?

If you would like more information on the topic of the use of aspirin for cancer prevention, I can email you an information sheet with website links with this further information. If you would like to ask me further questions on the study, please do not hesitate to contact me.

# Interview schedule for HCP interviews

Thank you for agreeing to take part. Just before we start the interview, I’d like to quickly go over some key points about your rights as a participant in this study. It is completely fine if at any point you would like to stop the interview, or if you do not want to answer a question, please just let me know. You can also withdraw from the study at any point and you do not have to provide a reason for why. Just to remind you the interview will be recorded, but anything you say to me today will be kept confidential, and if you are quoted in any of our reports your name will not be used and instead you will be given a pseudonym, which is a fake name. I will also anonymise anything identifiable, like places, you mention as well. After the interview, if you have any further questions about anything we discussed today, please feel free to contact me. I can also provide a debrief sheet at the end with website links with further information on this topic in case you are interested in reading more. Are you happy to continue?

## HCP interviews

As you read on the information sheet, the study is aiming to understand what healthcare providers think about the use of aspirin for cancer prevention, including the reasons why people may or may not be willing to recommend it. In the interview, we will go through a number of different scenarios and explore your potential responses to them. Each scenario describes a situation which you may encounter with patients with Lynch syndrome enquiring about the use of aspirin for cancer prevention. I would just like to emphasise before we start that no prior knowledge on the topic of aspirin for cancer prevention is needed or expected. We are just interested in exploring your initial reactions to these scenarios.

### General Practitioner (GP)

First, I would just like to ask what your initial thoughts are on the use of regular aspirin for cancer prevention?

Next, I would like to read the National Institute of Clinical Excellence (NICE) 2020 clinical guidelines for colorectal cancer [NG151] which states:

“Consider daily aspirin, to be taken for more than 2 years, to prevent colorectal cancer in people with Lynch syndrome.”

There is also a brief section on dosage in the NICE guidelines, which states:

“The optimal dose of aspirin that balances the benefits of aspirin in preventing colorectal cancer and the potential increased bleeding risk (especially with higher doses) remains unclear. Because of this the committee was not able to recommend a dose… Commonly used doses in current practice are 150mg or 300mg.”

I will now ask some interview questions to explore your views and attitudes towards this NICE guideline on daily aspirin for people with Lynch syndrome.

| **Domains** | **Interview questions** |
| --- | --- |
| Knowledge | Had you heard about the use of aspirin for cancer prevention, not just in a Lynch syndrome population, before this interview?  If yes, where and how did you hear about this?  Had you heard of the new NICE guideline on daily aspirin for people with Lynch Syndrome before this interview?  If yes, where and how did you hear about this NICE guideline?  What does the NICE guideline to ‘consider daily aspirin to prevent colorectal cancer in people with Lynch syndrome’ mean to you? Prompts: What do you think it is asking healthcare professionals to do? |
| Social/ Professional role and identity | What role do you see primary care playing in the implementation of this guidance on the use of aspirin for people with Lynch syndrome? |
| Skills  Reinforcement | What support do you think you need to implement this guidance in practice?  Do you have any previous experience of prescribing aspirin for cancer prevention? Is there anything about this experience which makes you more or less likely to prescribe aspirin for cancer prevention in the future? |
| Optimism  Enviromental context and resources  Beliefs about consequences | In your opinion, do you think regularly using aspirin is an effective way to reduce a patient with Lynch syndrome’s risk of developing colorectal cancer? What further information would you need?  Do you have any concerns about people taking aspirin regularly? Prompts: How do you feel about the side-effects? |

Imagine a situation where a patient with Lynch syndrome comes into their 10-minute appointment with you to ask about the use of aspirin for cancer prevention.

| Skills  Intentions  Beliefs about capabilties  Enviromental context and resources | Could you describe to me the first steps you might take in supporting this patient? Prompt: Why would you take these steps?  How confident would you feel discussing the use of aspirin for cancer prevention with this patient? Prompt: why is this? What do you think could help you overcome these problems?  Do you feel you have enough resources to support people with Lynch syndrome who are considering the use of aspirin for cancer prevention? Prompt: time, materials, training, support? What other resources do you think are needed? |
| --- | --- |
| Goals  Memory, attention and decision processes | Taking into consideration all the other things you could discuss in a typical consultation with a patient with Lynch syndrome, how important do you think discussing the use of aspirin for cancer prevention is? Prompt: Why do you feel this is more/ less important? |
| Social influences | How do the people you work with influence your decisions around whether to prescribe aspirin for cancer prevention? Prompt: colleagues in your practice team? Colleagues in secondary care? Clinical commissioning groups? Medicine management groups? |

Now I will describe a different scenario where you have received a letter from a clinical geneticist requesting for a patient of yours with Lynch syndrome to be prescribed daily aspirin for cancer prevention. The patient then comes into their GP appointment with you to obtain this prescription.

| Skills  Intentions  Beliefs about capabilties  Emotions | Taking the letter into consideration, could you describe to me the first steps you might take in supporting this patient? Prompt: Why would you take these steps?  How comfortable would you feel prescribing daily aspirin to this patient? Prompt: why is this? |
| --- | --- |
| Emotions | How comfortable would you feel prescribing regular aspirin to a patient who does not have Lynch syndrome but is interested in using aspirin for cancer prevention? Prompt: why is this? |

### Community pharmacists, clinical geneticists, genetic counsellors

First, I would just like to ask what your initial thoughts are on the use of aspirin for cancer prevention?

Next, I would like to read the National Institute of Clinical Excellence (NICE) 2020 clinical guidelines for colorectal cancer [NG151] which states:

“Consider daily aspirin, to be taken for more than 2 years, to prevent colorectal cancer in people with Lynch syndrome.”

There is also a brief section on dosage in the NICE guidelines, which states:

“The optimal dose of aspirin that balances the benefits of aspirin in preventing colorectal cancer and the potential increased bleeding risk (especially with higher doses) remains unclear. Because of this the committee was not able to recommend a dose… Commonly used doses in current practice are 150mg or 300mg.”

I will now ask some interview questions to explore your views and attitudes towards this NICE guideline on daily aspirin for people with Lynch syndrome.

| **Domains** | **Interview questions** |
| --- | --- |
| Knowledge | Had you heard about the use of aspirin for cancer prevention, not just in a Lynch syndrome population, before this interview?  If yes, where and how did you hear about this?  Had you heard of the new NICE guideline on daily aspirin for people with Lynch Syndrome before this interview?  If yes, where and how did you hear about this NICE guideline?  What does the NICE guideline to ‘consider daily aspirin to prevent colorectal cancer in people with Lynch syndrome’ mean to you? Prompts: What do you think it is asking healthcare professionals to do? |
| Social/ Professional role and identity | What role do you see [community pharmacists/ clinical geneticists/ genetic counsellors] playing in the implementation of this guidance on considering the use of daily aspirin for people with Lynch syndrome? |
| Skills | What support do you think you need to implement this guidance in practice? |
| Optimism  Enviromental context and resources  Beliefs about consequences | In your opinion, do you think regularly using aspirin is an effective way to reduce a patient with Lynch syndrome’s risk of developing colorectal cancer? What further information would you need?  Do you have any concerns about people taking aspirin regularly? Prompts: How do you feel about the side-effects? |

Imagine a situation where a patient with Lynch syndrome comes into an [appointment with you/ into the pharmacy you work at] to ask you about the use of aspirin for cancer prevention.

| Skills  Intentions  Beliefs about capabilties  Enviromental context and resources | Could you describe to me the first steps you might take in supporting this patient? Prompt: Why would you take these steps?  How confident would you feel discussing the use of aspirin for cancer prevention with this patient? Prompt: why is this? What do you think could help you overcome these problems?  Do you feel you have enough resources to support people with Lynch syndrome who are considering the use of aspirin for cancer prevention? Prompt: time, materials, training, support? What other resources do you think are needed? |
| --- | --- |
| Social influences | How do the people you work with influence your decisions around whether to recommend aspirin for cancer prevention? Prompts: colleagues in your practice team? Colleagues in secondary care? Clinical commissioning groups? Medicine management groups? |
| Goals  Memory, attention and decision processes | Taking into consideration all the other things you could discuss with a patient with Lynch syndrome, how important do you think discussing the use of aspirin for cancer prevention is? Prompt: Why do you feel this is more/ less important? |
| Emotions | How comfortable would you feel recommending regular aspirin use to a patient who does not have Lynch syndrome but is interested in using aspirin for cancer prevention? Prompt: why is this? |

## HCP brief quantitative questions

Thank you so much for your answers so far. Just before we end the interview, there are several brief demographic questions that I would just like to go through with you. If you do not want to answer one these questions, please let me know and we can skip it.

1. What is your age?
2. How would you describe your gender? Male; Female; Non-binary; Another Identity; Prefer not to say
3. How would you describe your ethnicity? (Examples include White British, Indian, Mixed – White and Black Caribbean, etc.)
4. What country in the UK do you live?
5. What is your profession?
6. How many years have you worked in your profession?
7. Do you know if you have you previously encountered any patients with Lynch syndrome in your work? If so, approximately how often, e.g. Daily, weekly, monthly, once or twice a year?

## Debrief

Thank you for all your help with answering my questions. We really appreciate your time and hope that it will be useful in the future when we are trying to support people when making a decision about whether to use aspirin for cancer prevention. Before we end, do you have any questions you would like to ask me?

If you would like more information on the topic of the use of aspirin for cancer prevention, I can email you an information sheet with website links with this further information. If you would like to ask me further questions on the study, please do not hesitate to contact me.
